# Variant‐gene pair in *silico* analyses and functional validation of LOAD GWAS loci in human brain‐relevant cell types identify *EGFR* as a target gene for potential drug repurposing

**DOI:** 10.1002/alz.087633

**Published:** 2025-01-03

**Authors:** Yuk Yee Leung, Pavel P. Kuksa, Jeffrey Cifello, Emily Greenfest‐Allen, Louisa Boateng, Shannon Laub, Natalia Tulina, Gerard D. Schellenberg, Alessandra Chesi, Li‐San Wang

**Affiliations:** ^1^ Department of Pathology and Laboratory Medicine, University of Pennsylvania Perelman School of Medicine, Philadelphia, PA USA; ^2^ Penn Neurodegeneration Genomics Center, Perelman School of Medicine, University of Pennsylvania, Philadelphia, PA USA; ^3^ Penn Neurodegeneration Genomics Center, Dept of Pathology and Laboratory Medicine, University of Pennsylvania, Philadelphia, PA USA; ^4^ Department of Pathology and Laboratory Medicine, Perelman School of Medicine, University of Pennsylvania, Philadelphia, PA USA; ^5^ University of Pennsylvania, Philadelphia, PA USA; ^6^ Penn Neurodegeneration Genomics Center, Department of Pathology and Laboratory Medicine, University of Pennsylvania Perelman School of Medicine, Philadelphia, PA USA

## Abstract

**Background:**

Recent studies suggest genome‐wide‐association‐studies (GWAS) loci confer their effects on microglia in late‐onset Alzheimer’s disease (LOAD) brains. Relatively fewer studies have investigated the effects of other genome‐wide significant loci (*p*<5e^‐8^) using human neurons.

**Method:**

GWAS itself cannot directly identify causal variant‐(effector)gene‐pairs as GWAS only reports the sentinel variant at a given locus. To map the physical interactions between GWAS‐implicated putative causal non‐coding variants and their corresponding effector genes in LOAD, we leveraged the post‐GWAS analytical framework, INFERNO, coupled with the largest harmonized functional genomics data catalog to date, FILER. Our goal was to unravel the effects of all 75 genome‐wide significant genetic loci reported in Bellenguez *et al* in human brain cell types. We searched for plausible evidence of causality for LOAD candidate variants in any brain‐specific variant‐gene pair through a total of 3,873 publicly curated datasets (123 enhancer, 3,153 epigenetics, 105 QTL) across 12 resources. We then validated the top variant‐gene pairs using high‐resolution promoter‐focused Capture C, ATAC‐seq and RNA‐seq datasets from several neuronal cell types and performed CRISPR interference.

**Result:**

The computational variant‐gene analyses identified a novel candidate causal SNP, rs74504435 (r2 = 0.94 to sentinel SNP rs76928645) at the *SEC61G* locus, located between *SEC61G/EGFR*. With a CADD score of 15, it is predicted as “regulatory” by both ENSEMBL and regulomeDB2. rs74504435 is found in ChromHMM and EpiMAP brain enhancers, and perturbs the transcriptional factor OCT6, which controls Schwann cell myelination. The minor allele T is protective to LOAD and downregulates *EGFR* in GTEx brain cortex (*p* = 2.3e^‐13^), CommonMind (FDR *p* = 5.8e^‐5^), and ROSMAP DLPFC (FDR *p* = 8.6e^‐9^) datasets with the same effect direction. In our Capture C and ATAC‐seq datasets, the SNP resides in open chromatin and directly contacts *EGFR* in human iPSC‐derived cortical neurons and ES‐derived hypothalamic neurons. We further validated a regulatory role for the enhancer region containing rs74504435 on *EGFR* using CRISPR interference in human microglial cells.

**Conclusion:**

Together, we identified a novel LOAD‐associated neuronal enhancer that drives *EGFR* gene expression. Further research is needed to investigate if *EGFR* inhibitors approved as anti‐cancer drugs can serve as potential candidates for repurposing as LOAD therapeutics.

